# Supplementary material for: Multilocus Genotyping of Giardia duodenalis in Mostly Asymptomatic Indigenous People from the Tapirapé Tribe, Brazilian Amazon
Source: Pathogens. 2021 Feb 14;10(2):206. doi: 10.3390/pathogens10020206 (PMC7917967; doi:10.3390/pathogens10020206)
Supplement: Supplementary file 1 [file pathogens-10-00206-s001.zip › pathogens-1056628-supplementary-final/pathogens-1056628-supplementary figures.docx]

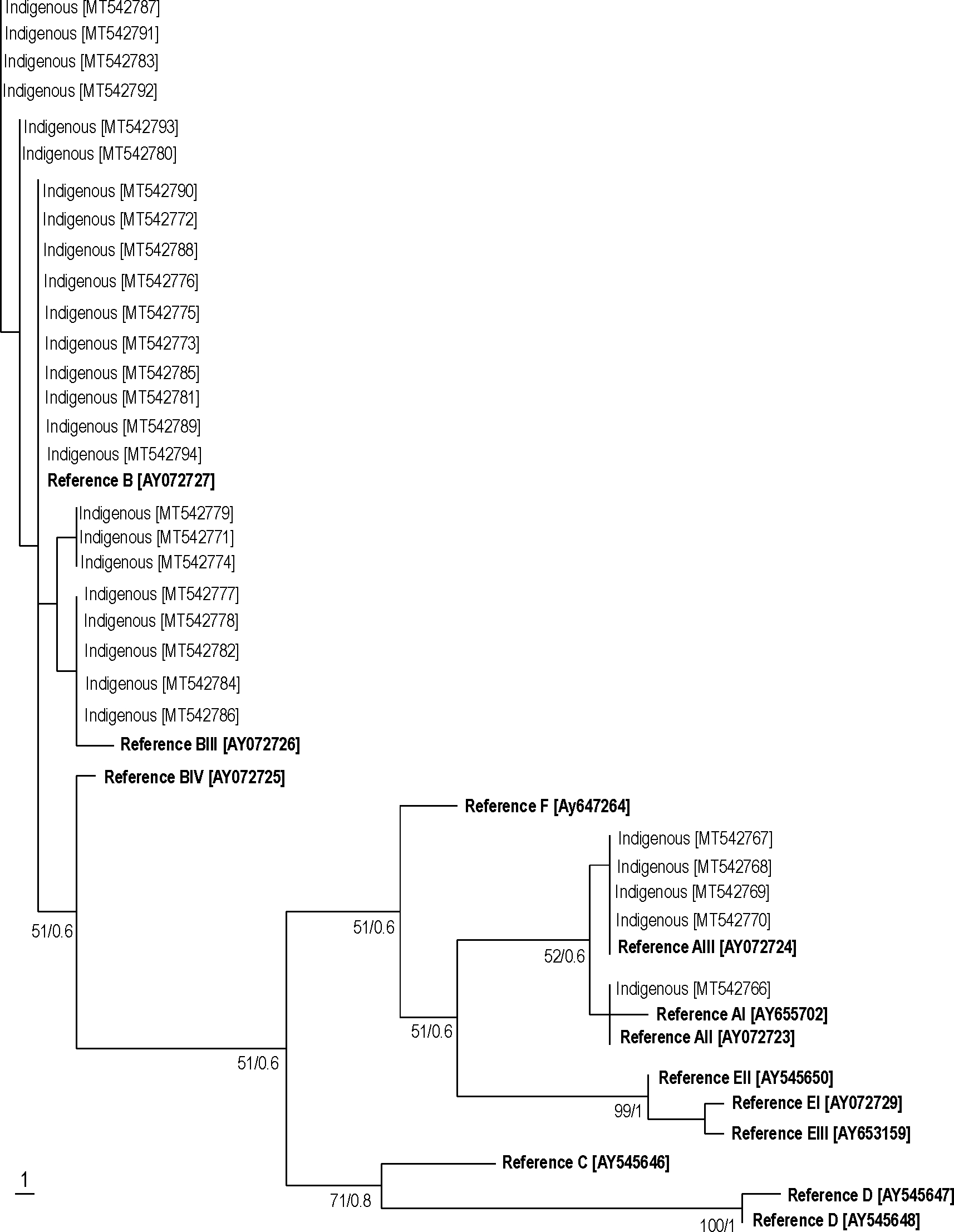


**Figure S1.** Maximum parsimony phylogenetic dendogram based on *bg* sequences of *G. duodenalis*. Numbers on nodes indicate the bootstrap/posterior probability values. GenBank accession numbers for all sequences used for the phylogenetic analysis were embedded in the tree.


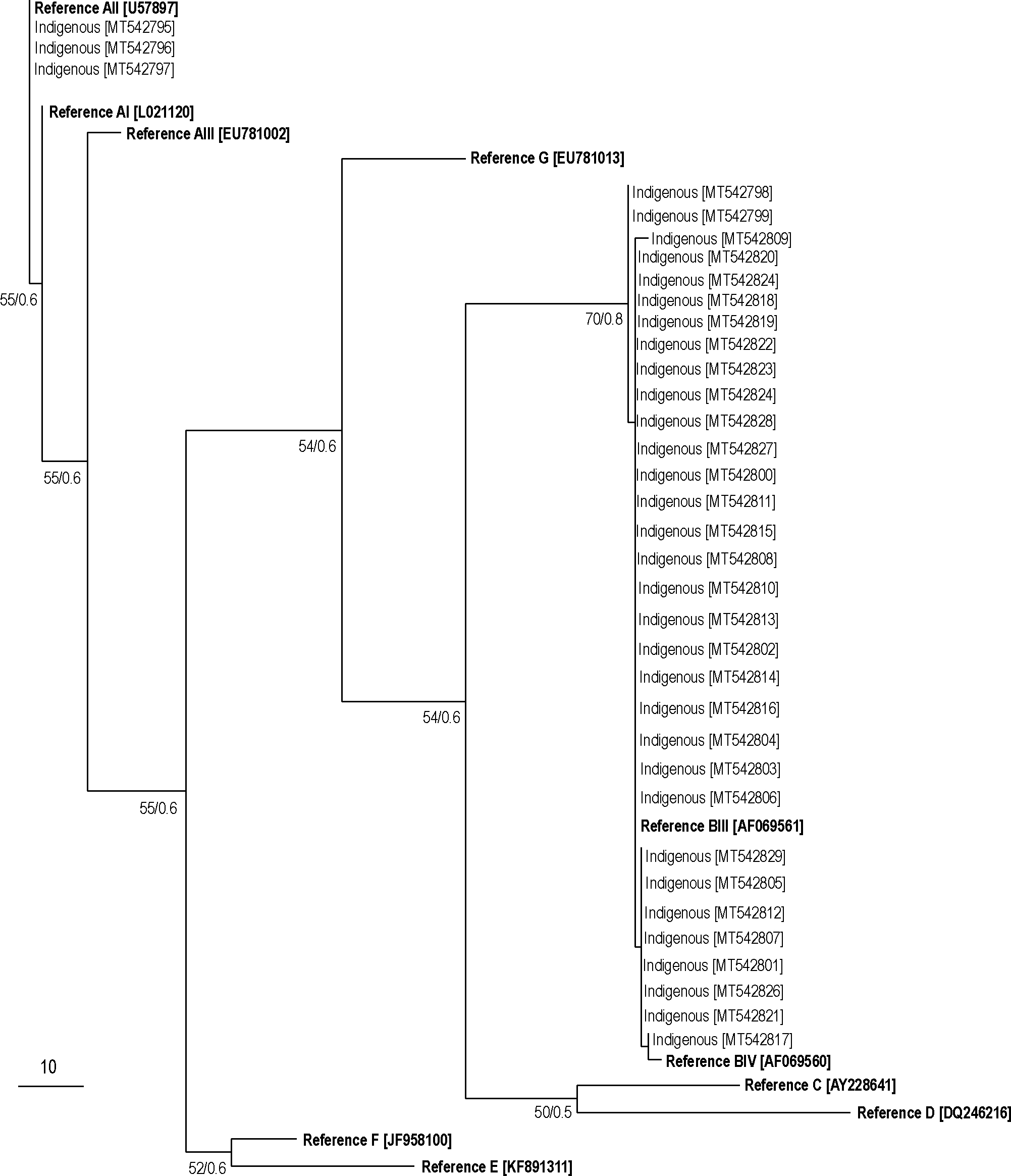


**Figure S2.** Maximum parsimony phylogenetic dendogram based on *tpi* sequences of *G. duodenalis*. Numbers on nodes indicate the bootstrap/posterior probability values. GenBank accession numbers for all sequences used for the phylogenetic analysis were embedded in the tree.
