# Supplementary material for: Multilocus Genotyping of Giardia duodenalis in Mostly Asymptomatic Indigenous People from the Tapirapé Tribe, Brazilian Amazon
Source: Pathogens. 2021 Feb 14;10(2):206. doi: 10.3390/pathogens10020206 (PMC7917967; doi:10.3390/pathogens10020206)
Supplement: Supplementary file 1 [file pathogens-10-00206-s001.zip › pathogens-1056628-supplementary-final/Table S1 Köster et al_Pathogens.docx]

**Table S1.** Prevalence and molecular diversity of *Giardia duodenalis* in humans in Brazil.

| **State** | **Period** | **Population** | **Samples (*n*)** | **Prevalence % (*n*/total)** | **Marker** | **Assemblage (%)** | **Sub-assemblage (%)** | **Reference** |
| --- | --- | --- | --- | --- | --- | --- | --- | --- |
| Amazonas | 2011 | Children | 433 | 17 (73/433) | *bg*, *gdh*, *tpi* | A (22), B (78) | AII, AIII, B^1^, BIII | [37] |
| Bahia | NS | Children | 39 | 100 (39/39)^2^ | *bg*, *gdh* | A (92), B (8) | AI^1^, AII, BIV | [65] |
| Ceará | 1989–93 | Children | 189 | 25 (47/189)^3^ | *ssu* rRNA | A (15), B (75), A+B (10) | Unknown | [27] |
|  | 2013 | Children | 213 | 14 (30/213) | *bg*, *gdh*, *tpi* | A (71), B (29) | AII^1^, B | [37] |
| Minas Gerais | 2007–08 | Children  Food handlers | 245  13 | 52 (127/245)  15 (2/13)^3^ | *gdh* | B (100)  B (100) | Unknown | [66] |
|  | 2013 | Children | 160 | 19 (30/160)^3^ | *bg*, *gdh*, *tpi* | A (67), B (14), A/B (19) | AII^1^, BIV, BIII/BIV, AII/BIV | [67] |
|  | NS | NS | 1 | 100 (1/1)^2^ | *ssu* rRNA | A (100) | Unknown | [68] |
|  | 2013 | Children | 66 | 23 (15/66)^3^ | *bg*, *gdh*, *tpi* | A (8), B (38), A/B (23), B/E (31) | AII, BIV^1^, BIII/BIV, AII/BIV | [67] |
| Paraná | 2010 | Residents | 134 | 18 (24/134)^3^ | *bg,* HSP | A (63), B (21), A/B (16) | Unknown | [69] |
|  | 2012–13 | Residents | 380 | 9 (34/380)^3^ | *bg*, *gdh* | A (26), B (74) | AI, AII, BIII, BIV^a^ | [70] |
|  | NS | Food handlers | 27 | 19 (5/27)^3^ | *bg*, *gdh* | A (25), B (75) | AII, BIV^1^ | [71] |
|  | 2015–16 | Residents | 766 | 11 (84/766) | *bg*, *gdh*, *ssu* rRNA | A (50), B (50) | AII^1^, AII/AIII, BIII, BIV, BIII/BIV | [41] |
| Piauí | 2014 | Children | 298 | 8 (25/298) | *bg*, *gdh*, *tpi* | A (50), B (50) | AII | [37] |
| Rio de Janeiro | 2003–05 | Children  Adults | 310  56 | 28 (86/310)^3^  2 (1/56)^3^ | *bg* | A (100) | AI^1^, AII | [72] |
|  | 2011–15 | Patients | 65 | 100 (65/65)^2^ | *bg*, *gdh*, *orf*C4, *ssu* rRNA, *tpi* | A (52), B (48) | AII^1^, BIII, BIV, BIII+BIV | [73] |
|  | 2011–15 | Patients HIV+  Patients HIV- | 38  27 | 100 (38/38)^2^  100 (27/27)^2^ | *bg*, *gdh*, *orf*C4, *ssu* rRNA, *tpi* | A (44), B (56)  A (65), B (35) | Unknown | [74] |
|  | 2011–15 | Patients | 65 | 100 (65/65)^2^ | *bg*, *gdh*, *tpi* | A (46), B (54) | AII^1^, BIII, BIV, BIII+BIV, BIII/BIV | [75] |
|  | 2015 | Children | 105 | 18 (19/105) | *bg*, *gdh* | A (58), B (32), E (10) | Unknown | [76] |
|  | NS | Children  Adults | 89  35 | 49 (44/89)  0.0 (0/35) | *bg*, *gdh* | A (66), E (34) | Unknown | [77] |
| Santa Catarina | 2010–11 | Children | 91 | 12 (11/91) | *gdh* | A (55), B (45) | AI, AII^1^, BIII, BIV | [78] |
| São Paulo | 2009–10 | General | 154 | 25 (39/154) | *bg* | A (100) | AI, AII/AIII^1^ | [79] |
|  | NS | Patients | 5 | 100 (5/5)^2^ | *gdh, tpi* | A (100) | AI, AII^1^ | [80] |
|  | 2012–13 | Children  Care workers  Relatives  Children^4^ | 123 14 44  19 | 34 (42/123)  0 (0/14)  32 (14/44)  37 (7/19) | *bg*, *gdh*, *tpi* | A (36), B (64)  B (100)  A (25), B (75) | AII, BIII/BIV, BIV^1^  BIV  AII, BIV^1^ | [40] |
|  | NS | Patients  Children | 51  28 | 100 (51/51)^2^  100 (28/28)^2^ | *bg*, *gdh*, *tpi* | A (42), B (42), C (6), A/B (6), A/C (4)  A (29), B (32), A/B (39) | AII^1^, BIII, BIV, BIII/BIV, AII/B  AII, BIII, BIV, AII/BIII, AII/BIV^1^ | [81] |
|  | 2017 | Children | 105 | 48 (50/105) | *bg*, *gdh*, *tpi* | A (60), B (38), A+B (2) | AII^1^, BIII, BIV | [82] |
|  | 2007–08 | Patients | 54 | 100 (54/54)^2^ | *bg* | A (100%) | AI^1^, AII | [83] |
|  | 2011–13 | General | 88 | 18 (16/88) | *bg*, *gdh*, *tpi* | A (47), B (53) | AI^1^, AII, BIV | [84] |
|  | 2011–13 | General | 28 | 8 (3/38) | *bg*, *gdh*, *tpi* | A (67), B (33) | AII^1^, BIV | [84] |
|  | 2004–06 | Patients | 37 | 100 (37/37)^2^ | *gdh* | A (78), B (22) | AII | [85] |

*bg*, β-giardin; *gdh*, Glutamate dehydrogenase; HIV, Human immunodeficiency virus; HSP, Heat shock protein; NS, No specified; *orf*C4, open reading frame C4; *ssu* rRNA, Small subunit ribosomal RNA; *tpi*, Triose phosphate isomerase. ^1^ Predominant sub-assemblage. ^2^ Previously identified as *G. duodenalis*-positive samples. ^3^ Prevalence of *G. duodenalis* estimated by using light microscopy. ^4^ Post-treatment.
