# Supplementary material for: Multilocus Genotyping of Giardia duodenalis in Mostly Asymptomatic Indigenous People from the Tapirapé Tribe, Brazilian Amazon
Source: Pathogens. 2021 Feb 14;10(2):206. doi: 10.3390/pathogens10020206 (PMC7917967; doi:10.3390/pathogens10020206)
Supplement: Supplementary file 1 [file pathogens-10-00206-s001.zip › pathogens-1056628-supplementary-final/Table S10 Köster et al_Pathogens.docx]

**Table S10.** Multivariable analysis comparing always *G. duodenalis*-negative results *vs*. always *G. duodenalis*-positive results. *p*-values marked in bold indicate numbers that are significant on the 95% confidence limit.

| **Variable** | **OR^1^** | **95% CI^1^** | ***P*-value** |
| --- | --- | --- | --- |
| Age group (years) |  |  |  |
| 0–4 | — | — |  |
| 5–9 | 0.24 | 0.06–0.89 | **0.041** |
| 10–14 | 0.17 | 0.03–0.68 | **0.018** |
| ≥15 | 0.05 | 0.01–0.20 | **<0.001** |
| Tribe |  |  |  |
| 1 | — | — |  |
| 2 | 0.26 | 0.01–1.51 | 0.2 |
| 3 | 0.00 |  | >0.9 |
| 4 | 0.37 | 0.02–2.18 | 0.4 |
| 5 | 5.55 | 1.61–19.4 | **0.006** |
| 6 | 0.00 |  | >0.9 |

^1^ OR = Odds ratio, CI = Confidence interval. *n* = 312 observations, removing 3 observations with missing values. AIC = 123.545.
