# Supplementary material for: Multilocus Genotyping of Giardia duodenalis in Mostly Asymptomatic Indigenous People from the Tapirapé Tribe, Brazilian Amazon
Source: Pathogens. 2021 Feb 14;10(2):206. doi: 10.3390/pathogens10020206 (PMC7917967; doi:10.3390/pathogens10020206)
Supplement: Supplementary file 1 [file pathogens-10-00206-s001.zip › pathogens-1056628-supplementary-final/Table S12 Köster et al_Pathogens.docx]

**Table S12.** Oligonucleotides used for the molecular identification and characterization of *Giardia duodenalis* in the present study.

| **Locus** | **Oligonucleotide** | **Sequence (5´–3´)** | **Reference** |
| --- | --- | --- | --- |
| *ssu* rRNA | Probe | FAM–CCCGCGGCGGTCCCTGCTAG–BHQ1 | [56] |
|  | Gd-80F | GACGGCTCAGGACAACGGTT | [56] |
|  | Gd-127R | TTGCCAGCGGTGTCCG | [56] |
| *gdh* | GDHeF | TCAACGTYAAYCGYGGYTTCCGT | [57] |
|  | GDHiF | CAGTACACCTCYGCTCTCGG | [57] |
|  | GDHiR | GTTRTCCTTGCACATCTCC | [57] |
| *bg* | G7_F | AAGCCCGACGACCTCACCCGCAGTGC | [58] |
|  | G759_R | GAGGCCGCCCTGGATCTTCGAGACGAC | [58] |
|  | G99_F | GAACGAACGAGATCGAGGTCCG | [58] |
|  | G609_R | CTCGACGAGCTTCGTGTT | [58] |
| *tpi* | AL3543 | AAATIATGCCTGCTCGTCG | [59] |
|  | AL3546 | CAAACCTTITCCGCAAACC | [59] |
|  | AL3544 | CCCTTCATCGGIGGTAACTT | [59] |
|  | AL3545 | GTGGCCACCACICCCGTGCC | [59] |

*bg*, β-giardin; *gdh*, Glutamate dehydrogenase; *ssu* rRNA, Small subunit ribosomal RNA; *tpi*, Triose phosphate isomerase.
