# Supplementary material for: Multilocus Genotyping of Giardia duodenalis in Mostly Asymptomatic Indigenous People from the Tapirapé Tribe, Brazilian Amazon
Source: Pathogens. 2021 Feb 14;10(2):206. doi: 10.3390/pathogens10020206 (PMC7917967; doi:10.3390/pathogens10020206)
Supplement: Supplementary file 1 [file pathogens-10-00206-s001.zip › pathogens-1056628-supplementary-final/Table S2 Köster et al_Pathogens.docx]

**Table S2.** Prevalence and molecular diversity of *Giardia duodenalis* in domestic and wildlife animal species in Brazil.

| **State** | **Period** | **Host** | **Samples (*n*)** | **Prevalence % (*n*/total)** | **Marker** | **Assemblage (%)** | **Sub-assemblage (%)** | **Reference** |
| --- | --- | --- | --- | --- | --- | --- | --- | --- |
| Minas Gerais | 2013–14 | Avian | 85 | 1 (1/85) | *ssu* rRNA | A (100) | Unknown | [86] |
|  | NS | Cattle  Sheep  Pig | 256  105  90 | 12 (30/256)^1^  25 (26/105)^1^  3 (3/90)^1^ | *gdh*, *tpi* | E (92), B (8)  E (75), E/A (12), E/B (13)  E (100) | BIII  AII^2^, BIII^2^ | [87] |
|  | NS | Dog | 97 | 20 (19/97)^1^ | *gdh*, *tpi* | D (12), D/A (25, D/B (38), D/C (19), D/E (6) | AI^2^, BIII, BIV | [88] |
| Paraná | 2012–13 | Dog  Cat | 32  2 | 9 (3/32)  0 (0/2) | *bg*, *gdh* | B (33), C (33), D (33) | BIV | [70] |
|  | NS | Dog | 200 | 12 (23/200) | *bg* | Unknown | Unknown | [89] |
| Rio de Janeiro | NS | Dog  Cat | 24  28 | 29 (7/24)  6 (1/18) | *bg*, *gdh*, *tpi* | Unknown | Unknown | [90] |
|  | 2003–05 | Dogs and a cat | 29 | 28 (8/29) | *bg* | A (100) | AI | [72] |
|  | 2009–12 | Dog | 60 | 100 (60/60)^3^ | *bg*, *gdh* | A (100) | Unknown | [91] |
| Santa Catarina | 2005–2006 | Howler monkey | 28 | 57 (16/28) | *bg* | A (100) | AI | [92] |
|  | 2010–11 | Dog | 108 | 9 (10/108) | *gdh* | A (50), B (30), C (20) | AI^2^, BIV | [78] |
| São Paulo | 2004–06 | Dog  Cat  Cattle | 27  19  5 | 100 (27/27)^3^  100 (19/19)^3^  100 (5/5)^3^ | *gdh* | C (26), D (74)  A (42), F (58)  A (20), E (80) | –  AI  AI | [85] |
|  | 2007–08 | Dog | 5 | 100 (5/5)^3^ | *bg* | A (5) (100) | AI^2^, AII | [83] |
|  | NS | Howler monkey  Chinchilla  Ostrich  Jaguar | 20  3  2  1 | 100 (20/20)^3^  100 (3/3)^3^  100 (2/2)^3^  100 (1/1)^3^ | *ssu* rRNA,  *gdh* | B (100)  B (100)  B (100)  A (1) (100) | Unknown  Unknown  Unknown  AI | [93] |
|  | 2016 | Buffalo | 183 | 7 (12/183) | *bg*, *gdh*, *tpi* | E (100) | – | [94] |
|  | 2009–10 | Dog  Cat  Goat  Sheep  Calf | 20  10  5  20  6 | 20 (4/20)^1^  20 (2/10)^1^  20 (1/5)^1^  30 (6/20)^1^  17 (1/6)^1^ | *bg* | A (100)  A (100)  Unknown  Unknown  Unknown | AI  AI  Unknown  Unknown  Unknown | [79] |
|  | 2009 | NHP | 22 | 9 (4/47) | *gdh*, *tpi* | A (3) (100) | AI/AII^2^, AII | [95] |
|  | 2008–09 | Dog (pet)  Dog (stray) | 160  140 | 6 (10/160)  30 (42/140) | *ssu* rRNA,  *gdh* | C (56), D (31), C+D (14) | – | [96] |
|  | 2006 | Cattle | 200 | 8 (15/200) | *ssu* rRNA,  *gdh* | A (7), E (93) | AI | [97] |
|  | NS | Sheep | 100 | 34 (34/100) | *ssu* rRNA,  *gdh* | E (100) | – | [98] |

*bg*, β-giardin (bg); *gdh*, Glutamate dehydrogenase; NHP, Non-human primates; NS, No specified; *ssu* rRNA, Small subunit ribosomal RNA; *tpi*, Triose phosphate isomerase. ^1^ Prevalence of *G. duodenalis* estimated by using light microscopy. ^2^ Predominant sub-assemblage. ^3^ Previously identified as *G. duodenalis*-positive samples.
