# Supplementary material for: Multilocus Genotyping of Giardia duodenalis in Mostly Asymptomatic Indigenous People from the Tapirapé Tribe, Brazilian Amazon
Source: Pathogens. 2021 Feb 14;10(2):206. doi: 10.3390/pathogens10020206 (PMC7917967; doi:10.3390/pathogens10020206)
Supplement: Supplementary file 1 [file pathogens-10-00206-s001.zip › pathogens-1056628-supplementary-final/Table S3 Köster et al_Pathogens.docx]

**Table S3.** Prevalence and molecular diversity of *Giardia duodenalis* in water samples in Brazil.

| **State** | **Period** | **Water type** | **Samples (*n*)** | **Prevalence % (*n*/total)** | **Marker** | **Assemblage (%)** | **Sub-assemblage (%)** | **Reference** |
| --- | --- | --- | --- | --- | --- | --- | --- | --- |
| Paraná | 2012–13 | Irrigation | 44 | 0 (0/44) | *bg*, *gdh* | – | – | [70] |
| Paraná | 2014–16 | Irrigation | 10 | 30 (3/10) | *ssu* rRNA, *gdh*, *tpi* | Unknown | Unknown | [99] |
|  | 2005 | Raw  Treated | 24  24 | 8.3 (2/24)  0.0 (2/24) | *ssu* rRNA | Unknown  – | Unknown  – | [100] |
|  | 2015 | Sludge | 33 | 6 (2/33) | *tpi* | Unknown | Unknown | [101] |
| Santa Catarina | 2009 | Seawater | 4 | 25 (1/4) | *bg* | A (1) (100) | Unknown | [102] |
| São Paulo | 2013 | Water catchments | 50 | 52 (26/50) | *gdh* | A (4) (100) | Unknown | [103] |
|  | 2012–13 | Wastewater | 23 | 65 (15/23) | *gdh* | A (46), B (27), A/B (27) | Unknown | [104] |
|  | NS | River, sewage, urban streams | 6 | 100 (6/6) | *bg*, *gdh*, *tpi* | A (17), B (50), C (17), B/D (16) | AII, BIII^1^  BIV, D/BIV | [81] |
|  | 2013–14 | Raw sewage | 144 | 6.9 (10/144) | *bg*, *gdh*, *tpi* | A (33), C (33), B (33) | AII, BIV | [105] |
|  | 2009–10 | Estuarine | NS | NS | *tpi* | A (80), C (20) | AII | [106] |
|  | 2008–09 | Raw sewage  Treated sewage  Surface  Springs | 6  6  11  1 | 83 (5/6)  67 (4/6)  36 (4/11)  0 (0/1) | *gdh* | A (60), B (20), A+B (20)  A (4) (100)  A (4) (100)  – | AII  AII  AII  – | [107] |

*bg*, β-giardin; *gdh*, Glutamate dehydrogenase; NS, No specified; PCR, Polymerase chain reaction; *ssu* rRNA, Small subunit ribosomal RNA; *tpi*, Triose phosphate isomerase. ^1^ Predominant sub-assemblage
