# Supplementary material for: Multilocus Genotyping of Giardia duodenalis in Mostly Asymptomatic Indigenous People from the Tapirapé Tribe, Brazilian Amazon
Source: Pathogens. 2021 Feb 14;10(2):206. doi: 10.3390/pathogens10020206 (PMC7917967; doi:10.3390/pathogens10020206)
Supplement: Supplementary file 1 [file pathogens-10-00206-s001.zip › pathogens-1056628-supplementary-final/Table S4 Köster et al_Pathogens.docx]

**Table S4.** Prevalence and molecular diversity of *Giardia duodenalis* in fresh produce in Brazil.

| **State** | **Period** | **Product** | **Samples (*n*)** | **Diagnostic method** | **Prevalence % (*n*/total)** | **Marker** | **Assemblage (%)** | **Sub-assemblage (%)** | **Reference** |
| --- | --- | --- | --- | --- | --- | --- | --- | --- | --- |
| Paraná | 2012–13 | Vegetables | 11 | PCR | 18 (2/11) | *bg*, *gdh* | B (100) | BIV | [70] |
|  | 2014 | Vegetables | 128 | IFA, PCR-RFLP | 12.5 (16/128) | *gdh* | A (100) | AII | [108] |
|  | 2015–16 | Vegetables | 260 | PCR-RFLP | 7 (19/260) | *gdh* | A (82), B (9), E (9) | AI | [109] |
| São Paulo | 2009–10 | Oysters | NS | IFA, PCR | NS | *tpi* | A (100) | AII | [106] |

*bg*, beta-giardin; *gdh*, glutamate dehydrogenase; IFA, immunofluorescence assay; NS, no specified; PCR, polymerase chain reaction; *tpi*, triose phosphate isomerase.
