# Supplementary material for: Multilocus Genotyping of Giardia duodenalis in Mostly Asymptomatic Indigenous People from the Tapirapé Tribe, Brazilian Amazon
Source: Pathogens. 2021 Feb 14;10(2):206. doi: 10.3390/pathogens10020206 (PMC7917967; doi:10.3390/pathogens10020206)
Supplement: Supplementary file 1 [file pathogens-10-00206-s001.zip › pathogens-1056628-supplementary-final/Table S7 Köster et al_Pathogens.docx]

**Table S7.** Univariable analysis comparing discontinuously *G. duodenalis*-positive results versus continuously *G. duodenalis*-positive results. *p*-values marked in bold indicate numbers that are significant on the 95% confidence limit.

|  | ***G. duodenalis* (discontinuously positive/always positive)** | | **Crude Association** | | | |
| --- | --- | --- | --- | --- | --- | --- |
| **Variable** | **Discontinuously positive, *n* = 163^1^** | **Always positive, *n* = 20^1^** | ***n*** | **OR^2^** | **95% CI^2^** | ***p*-value** |
| Sex |  |  | 183 |  |  |  |
| 0 | 95.0 (90.5%) | 10.0 (9.5%) |  | — | — |  |
| 1 | 68.0 (87.2%) | 10.0 (12.8%) |  | 1.40 | 0.54–3.59 | 0.5 |
| Age group (years) |  |  | 183 |  |  |  |
| 0–4 | 34.0 (77.3%) | 10.0 (22.7%) |  | — | — |  |
| 5–9 | 43.0 (91.5%) | 4.0 (8.5%) |  | 0.32 | 0.08–1.04 | 0.070 |
| 10–14 | 31.0 (91.2%) | 3.0 (8.8%) |  | 0.33 | 0.07–1.19 | 0.11 |
| ≥15 | 55.0 (94.8%) | 3.0 (5.2%) |  | 0.19 | 0.04–0.66 | **0.015** |
| Tribe |  |  | 183 |  |  |  |
| 1 | 89.0 (89.0%) | 11.0 (11.0%) |  | — | — |  |
| 2 | 18.0 (94.7%) | 1.0 (5.3%) |  | 0.45 | 0.02–2.54 | 0.5 |
| 3 | 12.0 (100.0%) | 0.0 (0.0%) |  | 0.00 |  | >0.9 |
| 4 | 7.0 (87.5%) | 1.0 (12.5%) |  | 1.16 | 0.06–7.42 | 0.9 |
| 5 | 23.0 (76.7%) | 7.0 (23.3%) |  | 2.46 | 0.83–6.99 | 0.093 |
| 6 | 14.0 (100.0%) | 0.0 (0.0%) |  | 0.00 |  | >0.9 |
| Faecal consistency |  |  | 183 |  |  |  |
| 1 | 37.0 (86.0%) | 6.0 (14.0%) |  | — | — |  |
| 2 | 72.0 (88.9%) | 9.0 (11.1%) |  | 0.77 | 0.26–2.45 | 0.6 |
| 3 | 6.0 (100.0%) | 0.0 (0.0%) |  | 0.00 |  | >0.9 |
| 4 | 48.0 (90.6%) | 5.0 (9.4%) |  | 0.64 | 0.17–2.29 | 0.5 |
| Faecal appearance |  |  | 183 |  |  |  |
| 1 | 150.0 (89.3%) | 18.0 (10.7%) |  | — | — |  |
| 2 | 13.0 (86.7%) | 2.0 (13.3%) |  | 1.28 | 0.19–5.15 | 0.8 |
| Abdominal pain |  |  | 183 |  |  |  |
| 0 | 79.0 (91.9%) | 7.0 (8.1%) |  | — | — |  |
| 1 | 84.0 (86.6%) | 13.0 (13.4%) |  | 1.75 | 0.68–4.86 | 0.3 |
| Vomit |  |  | 183 |  |  |  |
| 0 | 158.0 (89.3%) | 19.0 (10.7%) |  | — | — |  |
| 1 | 5.0 (83.3%) | 1.0 (16.7%) |  | 1.66 | 0.08–11.1 | 0.7 |
| Treated water |  |  | 183 |  |  |  |
| 0 | 157.0 (89.2%) | 19.0 (10.8%) |  | — | — |  |
| 1 | 6.0 (85.7%) | 1.0 (14.3%) |  | 1.38 | 0.07–8.68 | 0.8 |
| Hand washing |  |  | 183 |  |  |  |
| 0 | 90.0 (84.1%) | 17.0 (15.9%) |  | — | — |  |
| 1 | 73.0 (96.1%) | 3.0 (3.9%) |  | 0.22 | 0.05–0.68 | **0.018** |
| Washing fresh produce |  |  | 182 |  |  |  |
| 0 | 27.0 (81.8%) | 6.0 (18.2%) |  | — | — |  |
| 1 | 135.0 (90.6%) | 14.0 (9.4%) |  | 0.47 | 0.17–1.41 | 0.2 |
| Unknown | 1 | 0 |  |  |  |  |
| Eating with |  |  | 183 |  |  |  |
| 1 | 134.0 (87.0%) | 20.0 (13.0%) |  | — | — |  |
| 2 | 29.0 (100.0%) | 0.0 (0.0%) |  | 0.00 |  | >0.9 |
| Defecation place |  |  | 183 |  |  |  |
| 1 | 13.0 (92.9%) | 1.0 (7.1%) |  | — | — |  |
| 3 | 118.0 (92.9%) | 9.0 (7.1%) |  | 0.99 | 0.17–19.0 | >0.9 |
| 5 | 32.0 (76.2%) | 10.0 (23.8%) |  | 4.06 | 0.67–78.4 | 0.2 |
| Contact with animals |  |  | 183 |  |  |  |
| 0 | 26.0 (92.9%) | 2.0 (7.1%) |  | — | — |  |
| 1 | 137.0 (88.4%) | 18.0 (11.6%) |  | 1.71 | 0.45–11.2 | 0.5 |
| Albendazole |  |  | 183 |  |  |  |
| 0 | 156.0 (88.6%) | 20.0 (11.4%) |  | — | — |  |
| 1 | 7.0 (100.0%) | 0.0 (0.0%) |  | 0.00 |  | >0.9 |
| Mebendazole |  |  | 183 |  |  |  |
| 0 | 135.0 (88.8%) | 17.0 (11.2%) |  | — | — |  |
| 1 | 28.0 (90.3%) | 3.0 (9.7%) |  | 0.85 | 0.19–2.75 | 0.8 |
| Metronidazole |  |  | 183 |  |  |  |
| 0 | 1.0 (50.0%) | 1.0 (50.0%) |  | — | — |  |
| 1 | 162.0 (89.5%) | 19.0 (10.5%) |  | 0.12 | 0.00–3.05 | 0.14 |
| Praziquantel |  |  | 183 |  |  |  |
| 0 | 155.0 (89.1%) | 19.0 (10.9%) |  | — | — |  |
| 1 | 8.0 (88.9%) | 1.0 (11.1%) |  | 1.02 | 0.05–6.01 | >0.9 |
| Tiabendazole |  |  | 183 |  |  |  |
| 0 | 157.0 (89.2%) | 19.0 (10.8%) |  | — | — |  |
| 1 | 6.0 (85.7%) | 1.0 (14.3%) |  | 1.38 | 0.07–8.68 | 0.8 |
| *Ancylostom*a (any) |  |  | 183 |  |  |  |
| 0 | 132.0 (89.2%) | 16.0 (10.8%) |  | — | — |  |
| 1 | 31.0 (88.6%) | 4.0 (11.4%) |  | 1.06 | 0.29–3.15 | >0.9 |
| *Ascaris* (any) |  |  | 183 |  |  |  |
| 0 | 160.0 (88.9%) | 20.0 (11.1%) |  | — | — |  |
| 1 | 3.0 (100.0%) | 0.0 (0.0%) |  | 0.00 |  | >0.9 |
| *Blastocystis* (any) |  |  | 183 |  |  |  |
| 0 | 141.0 (88.7%) | 18.0 (11.3%) |  | — | — |  |
| 1 | 22.0 (91.7%) | 2.0 (8.3%) |  | 0.71 | 0.11–2.71 | 0.7 |
| *Chilomastix* (any) |  |  | 183 |  |  |  |
| 0 | 132.0 (88.0%) | 18.0 (12.0%) |  | — | — |  |
| 1 | 31.0 (93.9%) | 2.0 (6.1%) |  | 0.47 | 0.07–1.76 | 0.3 |
| *E. coli* (any) |  |  | 183 |  |  |  |
| 0 | 78.0 (84.8%) | 14.0 (15.2%) |  | — | — |  |
| 1 | 85.0 (93.4%) | 6.0 (6.6%) |  | 0.39 | 0.13–1.03 | 0.069 |
| *E. histolytica* (any) |  |  | 183 |  |  |  |
| 0 | 101.0 (87.1%) | 15.0 (12.9%) |  | — | — |  |
| 1 | 62.0 (92.5%) | 5.0 (7.5%) |  | 0.54 | 0.17–1.48 | 0.3 |
| *E. nana* (any) |  |  | 183 |  |  |  |
| 0 | 68.0 (82.9%) | 14.0 (17.1%) |  | — | — |  |
| 1 | 95.0 (94.1%) | 6.0 (5.9%) |  | 0.31 | 0.10–0.81 | **0.021** |
| *Hymenolepis* (any) |  |  | 183 |  |  |  |
| 0 | 151.0 (89.3%) | 18.0 (10.7%) |  | — | — |  |
| 1 | 12.0 (85.7%) | 2.0 (14.3%) |  | 1.40 | 0.21–5.68 | 0.7 |
| *Iodamoeba* (any) |  |  | 183 |  |  |  |
| 0 | 157.0 (88.7%) | 20.0 (11.3%) |  | — | — |  |
| 1 | 6.0 (100.0%) | 0.0 (0.0%) |  | 0.00 |  | >0.9 |
| *Isospora* (any) |  |  | 183 |  |  |  |
| 0 | 162.0 (89.0%) | 20.0 (11.0%) |  | — | — |  |
| 1 | 1.0 (100.0%) | 0.0 (0.0%) |  | 0.00 |  | >0.9 |
| *Sarcocystis* (any) |  |  | 183 |  |  |  |
| 0 | 162.0 (89.5%) | 19.0 (10.5%) |  | — | — |  |
| 1 | 1.0 (50.0%) | 1.0 (50.0%) |  | 8.53 | 0.33–222 | 0.14 |
| *Strongyloides* (any) |  |  | 183 |  |  |  |
| 0 | 158.0 (89.3%) | 19.0 (10.7%) |  | — | — |  |
| 1 | 5.0 (83.3%) | 1.0 (16.7%) |  | 1.66 | 0.08–11.1 | 0.7 |
| *Trichuris* (any) |  |  | 183 |  |  |  |
| 0 | 162.0 (89.0%) | 20.0 (11.0%) |  | — | — |  |
| 1 | 1.0 (100.0%) | 0.0 (0.0%) |  | 0.00 |  | >0.9 |
| *Cyclospora* (any) |  |  | 183 |  |  |  |
| 0 | 154.0 (89.0%) | 19.0 (11.0%) |  | — | — |  |
| 1 | 9.0 (90.0%) | 1.0 (10.0%) |  | 0.90 | 0.05–5.20 | >0.9 |
| nb_samples |  |  | 183 | 0.45 | 0.24–0.84 | **0.014** |
| 2 | 29.0 (78.4%) | 8.0 (21.6%) |  |  |  |  |
| 3 | 64.0 (88.9%) | 8.0 (11.1%) |  |  |  |  |
| 4 | 70.0 (94.6%) | 4.0 (5.4%) |  |  |  |  |

^1^ Statistics presented: *n* (%). ^2^ OR = Odds Ratio, CI = Confidence Interval. *n* = 183 observations with at least one positive *G. duodenalis* result and number of samples >1.
