# Supplementary material for: Multilocus Genotyping of Giardia duodenalis in Mostly Asymptomatic Indigenous People from the Tapirapé Tribe, Brazilian Amazon
Source: Pathogens. 2021 Feb 14;10(2):206. doi: 10.3390/pathogens10020206 (PMC7917967; doi:10.3390/pathogens10020206)
Supplement: Supplementary file 1 [file pathogens-10-00206-s001.zip › pathogens-1056628-supplementary-final/Table S8 Köster et al_Pathogens.docx]

**Table S8.** Multivariable analysis comparing discontinuously *G. duodenalis*-positive results versus always *G. duodenalis*-positive results. *p*-values marked in bold indicate numbers that are significant on the 95% confidence limit.

| **Variable** | **OR^1^** | **95% CI^1^** | ***p*-value** |
| --- | --- | --- | --- |
| nb_samples | 0.30 | 0.12–0.66 | **0.005** |
| Tribe |  |  |  |
| 1 | — | — |  |
| 2 | 0.29 | 0.01–1.96 | 0.3 |
| 3 | 0.00 |  | >0.9 |
| 4 | 0.35 | 0.02–3.31 | 0.4 |
| 5 | 4.76 | 1.30–18.6 | **0.019** |
| 6 | 0.00 |  | >0.9 |
| Age group (years) |  |  |  |
| 0–4 | — | — |  |
| 5–9 | 0.31 | 0.07–1.20 | 0.10 |
| 10–14 | 0.29 | 0.05–1.21 | 0.11 |
| ≥15 | 0.34 | 0.05–2.46 | 0.3 |
| Hand washing |  |  |  |
| 0 | — | — |  |
| 1 | 0.27 | 0.04–1.37 | 0.14 |

^1^ OR = Odds ratio, CI = Confidence interval. *n* = 182 observations, removing 1 observation with missing values.
