# Supplementary material for: Multilocus Genotyping of Giardia duodenalis in Mostly Asymptomatic Indigenous People from the Tapirapé Tribe, Brazilian Amazon
Source: Pathogens. 2021 Feb 14;10(2):206. doi: 10.3390/pathogens10020206 (PMC7917967; doi:10.3390/pathogens10020206)
Supplement: Supplementary file 1 [file pathogens-10-00206-s001.zip › pathogens-1056628-supplementary-final/Table S9 Köster et al_Pathogens.docx]

**Table S9.** Univariable analysis comparing always *G. duodenalis*-negative results versus always *G. duodenalis*-positive results. *P*-values marked in bold indicate numbers that are significant on the 95% confidence limit.

|  | ***G. duodenalis* (always neg./always pos.)** | | **Crude Association** | | | |
| --- | --- | --- | --- | --- | --- | --- |
| **Variable** | **0, *n* = 295^1^** | **1, *n* = 20^1^** | ***n*** | **OR^2^** | **95% CI^2^** | ***p*-value** |
| Sex |  |  | 315 |  |  |  |
| 0 | 152.0 (93.8%) | 10.0 (6.2%) |  | — | — |  |
| 1 | 143.0 (93.5%) | 10.0 (6.5%) |  | 1.06 | 0.42–2.66 | 0.9 |
| Age group (years) |  |  | 313 |  |  |  |
| 0–4 | 28.0 (73.7%) | 10.0 (26.3%) |  | — | — |  |
| 5–9 | 53.0 (93.0%) | 4.0 (7.0%) |  | 0.21 | 0.05–0.69 | **0.015** |
| 10–14 | 49.0 (94.2%) | 3.0 (5.8%) |  | 0.17 | 0.04–0.61 | **0.012** |
| ≥15 | 163.0 (98.2%) | 3.0 (1.8%) |  | 0.05 | 0.01–0.18 | **<0.001** |
| Unknown | 2 | 0 |  |  |  |  |
| Tribe |  |  | 315 |  |  |  |
| 1 | 132.0 (92.3%) | 11.0 (7.7%) |  | — | — |  |
| 2 | 41.0 (97.6%) | 1.0 (2.4%) |  | 0.29 | 0.02–1.57 | 0.2 |
| 3 | 36.0 (100%) | 0.0 (0.0%) |  | 0.00 |  | >0.9 |
| 4 | 44.0 (97.8%) | 1.0 (2.2%) |  | 0.27 | 0.01–1.46 | 0.2 |
| 5 | 18.0 (72.0%) | 7.0 (28.0%) |  | 4.67 | 1.55–13.5 | **0.005** |
| 6 | 24.0 (100%) | 0.0 (0.0%) |  | 0.00 |  | >0.9 |
| Faecal consistency |  |  | 315 |  |  |  |
| 1 | 82.0 (93.2%) | 6.0 (6.8%) |  | — | — |  |
| 2 | 158.0 (94.6%) | 9.0 (5.4%) |  | 0.78 | 0.27–2.39 | 0.6 |
| 3 | 9.0 (100%) | 0.0 (0.0%) |  | 0.00 |  | >0.9 |
| 4 | 46.0 (90.2%) | 5.0 (9.8%) |  | 1.49 | 0.41–5.19 | 0.5 |
| Faecal appearance |  |  | 315 |  |  |  |
| 1 | 269.0 (93.7%) | 18.0 (6.3%) |  | — | — |  |
| 2 | 26.0 (92.9%) | 2.0 (7.1%) |  | 1.15 | 0.18–4.29 | 0.9 |
| Abdominal pain |  |  | 314 |  |  |  |
| 0 | 145.0 (95.4%) | 7.0 (4.6%) |  | — | — |  |
| 1 | 149.0 (92.0%) | 13.0 (8.0%) |  | 1.81 | 0.72–4.93 | 0.2 |
| Unknown | 1 | 0 |  |  |  |  |
| Vomit |  |  | 315 |  |  |  |
| 0 | 285.0 (93.8%) | 19.0 (6.2%) |  | — | — |  |
| 1 | 10.0 (90.9%) | 1.0 (9.1%) |  | 1.50 | 0.08–8.47 | 0.7 |
| Treated water |  |  | 315 |  |  |  |
| 0 | 280.0 (93.6%) | 19.0 (6.4%) |  | — | — |  |
| 1 | 15.0 (93.8%) | 1.0 (6.2%) |  | 0.98 | 0.05–5.27 | >0.9 |
| Hand washing |  |  | 315 |  |  |  |
| 0 | 131.0 (88.5%) | 17.0 (11.5%) |  | — | — |  |
| 1 | 164.0 (98.2%) | 3.0 (1.8%) |  | 0.14 | 0.03–0.43 | **0.002** |
| Washing fresh produce |  |  | 315 |  |  |  |
| 0 | 54.0 (90.0%) | 6.0 (10.0%) |  | — | — |  |
| 1 | 241.0 (94.5%) | 14.0 (5.5%) |  | 0.52 | 0.20–1.53 | 0.2 |
| Eating with |  |  | 315 |  |  |  |
| 1 | 242.0 (92.4%) | 20.0 (7.6%) |  | — | — |  |
| 2 | 53.0 (100%) | 0.0 (0.0%) |  | 0.00 |  | >0.9 |
| Defecation place |  |  | 315 |  |  |  |
| 1 | 31.0 (96.9%) | 1.0 (3.1%) |  | — | — |  |
| 3 | 237.0 (96.3%) | 9.0 (3.7%) |  | 1.18 | 0.21–22.1 | 0.9 |
| 5 | 27.0 (73.0%) | 10.0 (27.0%) |  | 11.5 | 2.01–218 | **0.024** |
| Contact with animals |  |  | 315 |  |  |  |
| 0 | 43.0 (95.6%) | 2.0 (4.4%) |  | — | — |  |
| 1 | 252.0 (93.3%) | 18.0 (6.7%) |  | 1.54 | 0.42–9.88 | 0.6 |
| *Ancylostoma* (any) |  |  | 315 |  |  |  |
| 0 | 229.0 (93.5%) | 16.0 (6.5%) |  | — | — |  |
| 1 | 66.0 (94.3%) | 4.0 (5.7%) |  | 0.87 | 0.24–2.46 | 0.8 |
| *Ascaris* (any) |  |  | 315 |  |  |  |
| 0 | 293.0 (93.6%) | 20.0 (6.4%) |  | — | — |  |
| 1 | 2.0 (100%) | 0.0 (0.0%) |  | 0.00 |  | >0.9 |
| *Blastocystis* (any) |  |  | 315 |  |  |  |
| 0 | 246.0 (93.2%) | 18.0 (6.8%) |  | — | — |  |
| 1 | 49.0 (96.1%) | 2.0 (3.9%) |  | 0.56 | 0.09–2.02 | 0.4 |
| *Chilomastix* (any) |  |  | 315 |  |  |  |
| 0 | 249.0 (93.3%) | 18.0 (6.7%) |  | — | — |  |
| 1 | 46.0 (95.8%) | 2.0 (4.2%) |  | 0.60 | 0.09–2.18 | 0.5 |
| *E. coli* (any) |  |  | 315 |  |  |  |
| 0 | 117.0 (89.3%) | 14.0 (10.7%) |  | — | — |  |
| 1 | 178.0 (96.7%) | 6.0 (3.3%) |  | 0.28 | 0.10–0.72 | **0.012** |
| *E. histolytica* (any) |  |  | 315 |  |  |  |
| 0 | 179.0 (92.3%) | 15.0 (7.7%) |  | — | — |  |
| 1 | 116.0 (95.9%) | 5.0 (4.1%) |  | 0.51 | 0.16–1.37 | 0.2 |
| *E. nana* (any) |  |  | 315 |  |  |  |
| 0 | 97.0 (87.4%) | 14.0 (12.6%) |  | — | — |  |
| 1 | 198.0 (97.1%) | 6.0 (2.9%) |  | 0.21 | 0.07–0.54 | **0.002** |
| *Hymenolepis* (any) |  |  | 315 |  |  |  |
| 0 | 270.0 (93.8%) | 18.0 (6.2%) |  | — | — |  |
| 1 | 25.0 (92.6%) | 2.0 (7.4%) |  | 1.20 | 0.18–4.49 | 0.8 |
| *Iodamoeba* (any) |  |  | 315 |  |  |  |
| 0 | 269.0 (93.1%) | 20.0 (6.9%) |  | — | — |  |
| 1 | 26.0 (100%) | 0.0 (0.0%) |  | 0.00 |  | >0.9 |
| *Sarcocystis* (any) |  |  | 315 |  |  |  |
| 0 | 289.0 (93.8%) | 19.0 (6.2%) |  | — | — |  |
| 1 | 6.0 (85.7%) | 1.0 (14.3%) |  | 2.54 | 0.13–15.9 | 0.4 |
| *Strongyloides* (any) |  |  | 315 |  |  |  |
| 0 | 278.0 (93.6%) | 19.0 (6.4%) |  | — | — |  |
| 1 | 17.0 (94.4%) | 1.0 (5.6%) |  | 0.86 | 0.05–4.56 | 0.9 |
| *Taenia* (any) |  |  | 315 |  |  |  |
| 0 | 293.0 (93.6%) | 20.0 (6.4%) |  | — | — |  |
| 1 | 2.0 (100%) | 0.0 (0.0%) |  | 0.00 |  | >0.9 |
| *Trichuris* (any) |  |  | 315 |  |  |  |
| 0 | 294.0 (93.6%) | 20.0 (6.4%) |  | — | — |  |
| 1 | 1.0 (100%) | 0.0 (0.0%) |  | 0.00 |  | >0.9 |
| *Cyclospora* (any) |  |  | 315 |  |  |  |
| 0 | 281.0 (93.7%) | 19.0 (6.3%) |  | — | — |  |
| 1 | 14.0 (93.3%) | 1.0 (6.7%) |  | 1.06 | 0.06–5.71 | >0.9 |
| nb_samples |  |  | 315 | 0.85 | 0.45–1.56 | 0.6 |
| 2 | 99.0 (92.5%) | 8.0 (7.5%) |  |  |  |  |
| 3 | 129.0 (94.2%) | 8.0 (5.8%) |  |  |  |  |
| 4 | 67.0 (94.4%) | 4.0 (5.6%) |  |  |  |  |

^1^ Statistics presented: *n* (%). ^2^ OR = Odds Ratio, CI = Confidence Interval.
